# Draft genome sequence of traditional fermented bamboo shoot-origin *Levilactobacillus zymae* isolate obtained from India

**DOI:** 10.1128/mra.01075-25

**Published:** 2025-11-24

**Authors:** Moirangthem Goutam Singh, Diyashree Karmakar, Kumaraswamy Jeyaram, Romi Wahengbam

**Affiliations:** 1 Biological Sciences and Technology Division, CSIR - North East Institute of Science and Technology 81721, Jorhat, Assam, India; 2 Biological Sciences Division, Faculty of Science, Academy of Scientific and Innovative Research 550336, Ghaziabad, Uttar Pradesh, India; 3 Institute of Bioresources and Sustainable Development, Sikkim Centre 63565 , Gangtok, Sikkim, India; University of Maryland School of Medicine, Baltimore, Maryland, USA

**Keywords:** draft genome, whole genome sequencing, *Levilactobacillus zymae*, traditional fermented bamboo shoot, carbohydrate-active enzyme

## Abstract

The genome of *Levilactobacillus zymae* H2S4L2, isolated from an Indian traditional fermented bamboo shoot, was sequenced using Illumina MiSeq. The 2,750,751 bp genome had 53.18% GC content with 2,656 protein-coding genes, 4 rRNAs, and 58 tRNAs. Genome analysis may reveal adaptation in the fermentation ecosystem and health applications.

## ANNOUNCEMENT

Traditional ethnic fermented foods typically undergo spontaneous fermentation driven by diverse autochthonous microbial communities characterized by species succession (1). Traditional fermented bamboo shoot harbors lactic acid bacteria-dominated community composed of diverse multi-strain species unique to the fermentation ecosystem (2, 3). Notably, *Levilactobacillus zymae* is prevalent in traditional bamboo shoot fermentation in Northeast India (3). Here, we report the draft genome of *L. zymae* H2S4L2 (= MRC 21527) isolated from a 52-day fermentation sample of traditional fermented bamboo shoot, *hikhu*, from Ziro village (N27°35′ E93°49′, 1571 msl) in Northeast India, collected in November 2011. It was isolated in MRS agar (pH 5.7 ±  0.2) supplemented with 0.05% cysteine-HCl, 1% CaCO_3_, 1% maltose, and 1% fructose at 30°C for 72 h under O_2_-depleted and CO₂-enriched environment, and identified as *L. zymae* by 16S rRNA gene sequencing as described elsewhere (4). Genome analysis could reveal its adaptation in bamboo shoot fermentation and potential health applications.

Genomic DNA was extracted using NucleoMag Pathogen kit (Macherey-Nagel, Germany) from a 48 h-old culture of a single colony grown in MRS broth at 30°C under strict anaerobic conditions (80% N_₂_, 10% CO_₂_, and 10% H_₂_) in A35 anaerobic workstation (Don Whitley Scientific, UK). Paired-end (PE) library prepared using Illumina Nextera XT kit was sequenced in Illumina MiSeq to generate 2 × 150 bp PE reads. Quality assessment was performed using FastQC v0.12.1 (5). Low-quality reads were filtered using Trim_galore v0.6.10 (6). *De novo* assembly was performed using SPADES v3.13.1 (7). Assembly quality was assessed with QUAST v5.0.2 (8). Genome was annotated using the NCBI Prokaryotic Genome Annotation Pipeline (PGAP) v6.10 (9). Genome quality was assessed with CheckM2 v1.1.0 (10), and coverage was calculated using BWA v0.7.17-r1188, SAMtools v1.3.1, and BEDTools genomecov v2.27.1 (11–13). Default parameters were used for all software unless otherwise specified.

**Fig 1:**
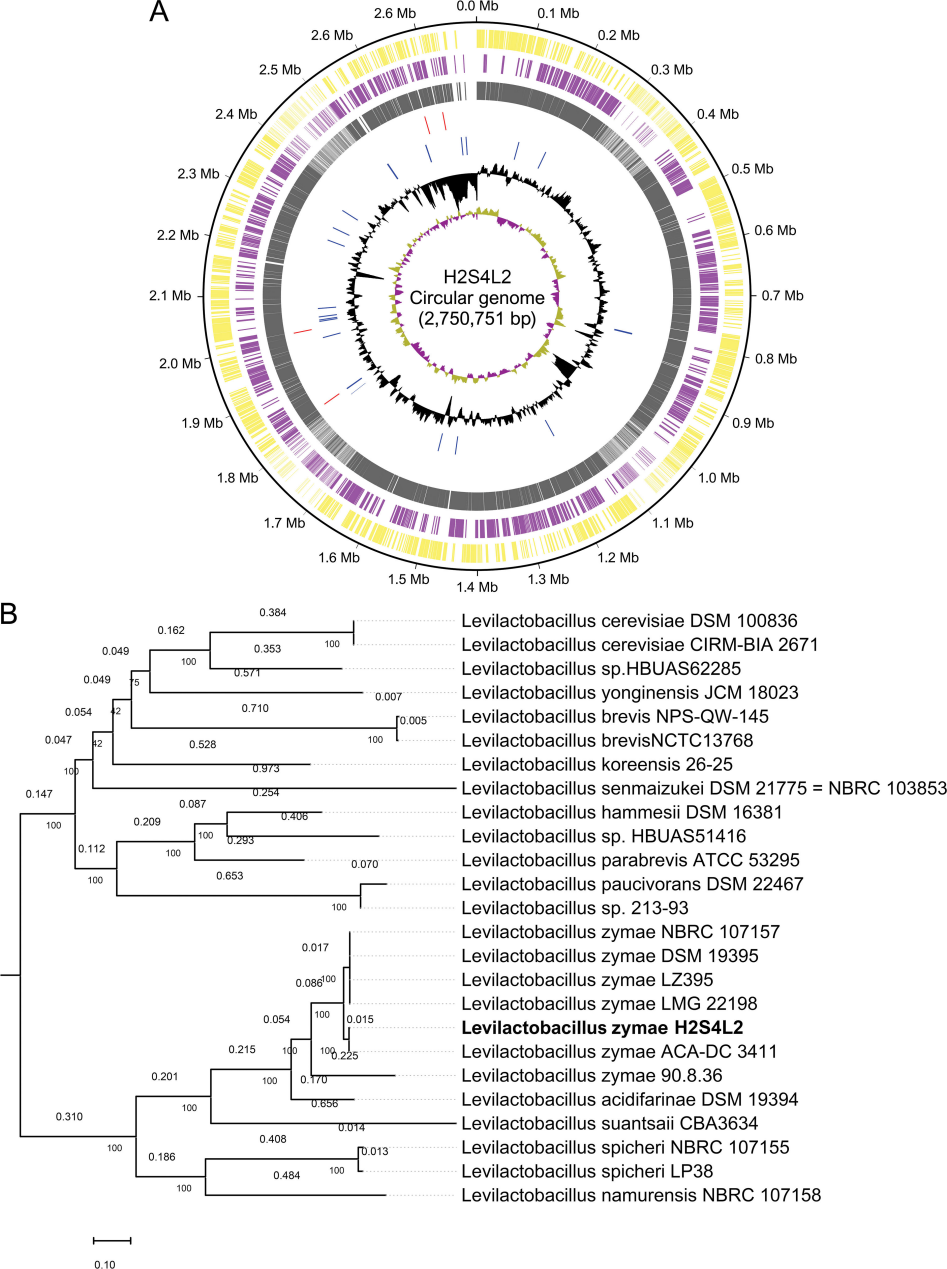
Circular genome visualization and phylogenomic relationship of *Levilactobacillus zymae* isolate H2S4L2. (**A**) Circular genome map showing distribution of the contigs with genome-mapped annotations. The concentric tracks of the visualization from inside to outside represent annotated genomic features: 1st track: GC skew showing +ve skew (green) and -ve skew (purple); 2nd track: GC content; 3rd track: tRNA, 4th track: rRNA, 5th track: protein-coding sequences (CDS); 6th track: open reading frame (ORF) on the reverse strand; and 7th track: ORF on the forward strand. (**B**) Phylogenomic tree constructed using 100 genes, showing the relationship of H2S4L2 with other strains from the genus *Levilactobacillus*. The reference genome LMG 22198 and five other genomes of *L. zymae* strains were included. The closest relative for the isolate under study is ACA-DC 3411, which is the first complete genome of *L. zymae* isolated from traditional Greek wheat sourdough. Values above the branches indicate evolutionary distances >0.001, and numbers below the branches near the node represent bootstrap percentage of how often the associated genome clustered together in replicate trees. The scale bar indicates evolutionary distance measured as the number of substitutions per site.

A total of 3,105,352 PE reads were trimmed, producing 2,906,570 high-quality (Q ≥ 30) PE reads. The genome size was 2,750,751 bp with 224-fold coverage, 53.18% GC content, and an N_50_ value of 31,454 bp. It consisted of 364 contigs with 2,656 coding sequences, 4 rRNAs,, and 58 tRNAs (Fig. 1A). CheckM2 identified 100% completeness and 0.36% contamination. Phylogenomic analysis using BV-BRC v3.50.5 (14) identified *L. zymae* ACA-DC 3411 as the closest known relative (Fig. 1B). Comparative genome analysis with the existing six *L. zymae* genomes using OrthoANIu USEARCH v11.0.667 (15) revealed >99% similarity with ACA-DC 3411. H2S4L2 had a single copy of rRNA operon (*rrn*), while other strains carried 5–6 *rrn* copies, highlighting potential evolutionary and functional differences. gutSMASH v2.0.0 (16) revealed core metabolic gene clusters for gallic acid metabolism, highlighting its implication in short-chain fatty acid biosynthesis (17). ResFinder v4.7.2 (18) revealed no antibiotic resistance genes, and PathogenFinder2 v0.5.0 (19) indicated a probability of 0.0529 for human pathogenicity, implicating its safety for human health. CAZyme annotation using dbCAN3 HMMdb v13 (20) identified 3 clusters comprising 61 genes across 17 glycoside hydrolase, 8 glycosyltransferase, and 4 carbohydrate esterase families, implicating its potential for complex carbohydrate metabolism and cyanogenic glycoside degradation in bamboo shoot fermentation.

## Data Availability

This Whole Genome Shotgun sequencing project has been deposited in DDBJ/ENA/NCBI GenBank under the accession no. JBPWQU000000000. The genome assembly version described in this paper is version JBPWQU010000000. All raw reads were deposited in the NCBI’s Sequence Read Archive under the accession no. SRR34674011. This project is associated with BioProject PRJNA1295091. The 16S rRNA gene sequence was deposited in NCBI GenBank under the accession no. KT757224.1.
